# Zika virus in the testes: should we be worried?

**DOI:** 10.1007/s13238-016-0357-3

**Published:** 2017-01-21

**Authors:** Gary Wong, Shihua Li, Lei Liu, Yingxia Liu, Yuhai Bi

**Affiliations:** 1 Shenzhen Key Laboratory of Pathogen and Immunity, State Key Discipline of Infectious Disease, Shenzhen Third People’s Hospital, Shenzhen, 518112 China; 2 CAS Key Laboratory of Pathogenic Microbiology and Immunology, Institute of Microbiology, Center for Influenza Research and Early-warning (CASCIRE), Chinese Academy of Sciences, Beijing, 100101 China

Zika virus (ZIKV) is a mosquito-borne pathogen from the family *Flaviviridae*, and infection of humans with ZIKV through mosquito bites may result in a disease known as Zika fever. Most Zika fever cases are asymptomatic and thus go unreported, but if symptoms do appear, they are usually mild in nature (fever, joint pain, headache and maculopapular rash), and self-resolve within one week (Chen and Hamer 2016). No deaths have ever been attributed to Zika fever; however, when a woman is infected with ZIKV during pregnancy, vertical transmission of the virus may result in microcephaly in the offspring (Rasmussen, Jamieson et al., 2016). ZIKV infections in adults are associated with episodes of Guillain-Barré syndrome, an autoimmune disorder of the peripheral nervous system, resulting in rapid-onset muscle weakness (Cao-Lormeau, Blake et al. 2016).

ZIKV was first isolated from a rhesus macaque in Uganda during 1947. Subsequent serological surveys showed that humans in Africa have long been exposed to the virus and many are seropositive for ZIKV-specific antibodies, suggesting that the virus had circulated amongst the human population for a considerable length of time (Dick, Kitchen et al. 1952). Until 2007, evidence of ZIKV infection in humans was observed only in African countries and Southeast Asia, with 14 confirmed cases reported during this time (Kindhauser, Allen et al. 2016). In 2007, an outbreak of Zika fever was reported in Yap Island, Federated States of Micronesia, which is the first outside Africa/Asia. There were 108 confirmed/suspect cases, but no hospitalizations or deaths (Duffy, Chen et al. 2009). Due to the minimal number of infections and the self-limiting nature of the disease, ZIKV was seen as an obscure pathogen of low public health priority and little scientific progress was made regarding this virus.

This changed when an outbreak of Zika fever began in Brazil in April 2015. Unlike previous instances, the virus spread rapidly to other countries throughout the Americas and the Caribbean, and has impacted 75 countries/territories as of November 2016 (WHO.int 2016). In particular, Singapore and Malaysia have both reported instances of locally-transmitted infections since August-September 2016 (MOH.gov.sg 2016). Meanwhile, China has reported over 20 cases of imported infections with ZIKV as of November 2016, and the viruses were shown to be highly genetically diverse (Zhang, Chen et al. 2016) (Shi, Zhang et al. 2016). Currently, over 100,000 people have been infected with ZIKV during this outbreak, and previously-unknown aspects of ZIKV infections are being reported as a result. For instance, male-to-female (Turmel, Abgueguen et al. 2016), male-to-male (Deckard, Chung et al. 2016) and female-to-male (Davidson, Slavinski et al. 2016) sexual transmission of ZIKV, a first for flavivirus infections, were all reported during this outbreak. Furthermore, ZIKV was observed to persist for at least six months in the semen of male patients after acute infection (Nicastri, Castilletti et al. 2016).

To characterize the nature of ZIKV infection in the male reproductive system, studies were published in *Nature* (Govero, Esakky et al. 2016) and *Cell* (Ma, Li et al. 2016) during October and November 2016, respectively. The studies showed that infection of (knockdown or knockout) Type I interferon (IFN) receptor-deficient mice with ZIKV resulted in chronic inflammation of the testes and epididymides (Govero, Esakky et al. 2016) (Ma, Li et al. 2016). Importantly, the virus persisted for weeks in the male reproductive tract after the initial infection, and led to an observable decrease in testes size (Govero, Esakky et al. 2016) (Ma, Li et al. 2016). A substantial reduction in sperm counts and fertility was observed in the *Nature* study (Govero, Esakky et al. 2016), while the *Cell* study showed that the mice have become infertile due to a complete destruction in testes morphology and the loss of stem-like cells (peritubular myoid cells and spermatogonia) (Ma, Li et al. 2016). Both studies also showed that this phenomenon was specific to ZIKV, as infection of mice with the closely-related Dengue virus (DENV) at similar or higher titers only resulted in transient, reversible damage to the male reproductive organs (Govero, Esakky et al. 2016) (Ma, Li et al. 2016).

What do these results mean from a public health perspective? While the results of these studies are no doubt concerning, they are not a cause for panic. It should be kept in mind that these findings, while novel and unexpected, are results derived from experiments with immunosuppressed mice. In the *Nature* paper, a mouse-adapted ZIKV (i.e. a strain that replicates well in mice) was used, in conjunction with IFN receptor-specific antibodies to weaken the host immune response (Govero, Esakky et al. 2016). In the *Cell* paper, while a clinical isolate of ZIKV was used, *Ifnar1* ^−*/*−^ mice were used to observe the results (Ma, Li et al. 2016). Damage to the male reproductive system was only observed in wild-type, immunocompetent mice when the virus was directly injected into the rete testis, which is not a natural route of infection with ZIKV. Importantly, while mice with a suppressed Type I IFN response developed symptoms of the central nervous system (i.e. paralysis) and died between 7–10 days after a challenge with ZIKV (Lazear, Govero et al. 2016), humans do not develop severe disease from ZIKV infection. Therefore, it can be argued that observations from knockout mice experiments may not necessarily correlate well with those in humans. The strongest argument against panic after infection with ZIKV is that there have been no reports thus far demonstrating or suspecting infertility in convalescent male human patients.

What these results do mean, however, is the need to broaden and deepen our understanding of ZIKV pathogenesis, but in a way that is more relatable to humans. The first step to achieve this would be to develop an animal model that more accurately recapitulates important aspects of clinical ZIKV disease as observed in humans. In a previous study, a group inoculated rhesus and cynomolgus macaques subcutaneously with ZIKV. While the animals showed no signs of disease, the virus was detected in the blood, urine, cerebrospinal fluid, semen and saliva, and transiently in vaginal fluid (Osuna, Lim et al. 2016). The virus was cleared from urine 10 days after infection, but was still detectable in saliva and seminal fluids at 4 weeks after infection (and almost 3 weeks after the resolution of viremia) (Osuna, Lim et al. 2016). These findings are in agreement with those from another study with rhesus macaques (Li, Dong et al. 2016). Overall, the observations from studies in nonhuman primates appear to be more consistent with the current clinical knowledge on Zika fever in humans. Therefore, these results (and any future unexpected findings in mice) should at least be characterized in nonhuman primates to assess the extent of damage to the host by ZIKV, and based on experimental data in conjunction with current clinical findings, re-evaluate the real threat posed by ZIKV infections to humans.
